# Supplementary material for: Meta-Analysis and Gene Set Enrichment Relative to ER Status Reveal Elevated Activity of MYC and E2F in the “Basal” Breast Cancer Subgroup
Source: PLoS One. 2009 Mar 9;4(3):e4710. doi: 10.1371/journal.pone.0004710 (PMC2650420; doi:10.1371/journal.pone.0004710)
Supplement: Dataset S1 — GSEA results from first screen. The file contains a zipped archive of the HTML results of the second GSEA screen. Reports for enriched or depleted datasets may be accessed from the “index.html” link within the my_analysis.GseaPreranked.1219975950625.rpt.zip ZIP archive. Enrichment plots are available for each of the 50 most enriched and depleted gene sets. (13.32 MB ZIP) [file pone.0004710.s001.zip › my_analysis.GseaPreranked.1219975950625.rpt.zip!//HDACI_COLON_TSABUT_UP.html]

Details for gene set HDACI\_COLON\_TSABUT\_UP[GSEA]

|  || Dataset | zSco.ERneg.to.ERpos\_collapsed |
| Phenotype | NoPhenotypeAvailable |
| Upregulated in class | na\_neg |
| GeneSet | HDACI\_COLON\_TSABUT\_UP |
| Enrichment Score (ES) | -0.42805234 |
| Normalized Enrichment Score (NES) | -1.8091823 |
| Nominal p-value | 0.0 |
| FDR q-value | 0.03535654 |
| FWER p-Value | 0.74 |
Table: GSEA Results Summary

  

Fig 1: Enrichment plot: HDACI\_COLON\_TSABUT\_UP      
 Profile of the Running ES Score & Positions of GeneSet Members on the Rank Ordered List

  

| PROBE | GENE SYMBOL | GENE\_TITLE | RANK IN GENE LIST | RANK METRIC SCORE | RUNNING ES | CORE ENRICHMENT || 1 | LRP8 |  |  | 163 | 5.498 | 0.0309 | No |
| 2 | SAT1 |  |  | 514 | 4.322 | 0.0384 | No |
| 3 | ETV5 |  |  | 1073 | 3.387 | 0.0229 | No |
| 4 | PLCB4 |  |  | 1692 | 2.765 | -0.0020 | No |
| 5 | FKBP5 |  |  | 1729 | 2.743 | 0.0168 | No |
| 6 | SAMD4A |  |  | 2082 | 2.462 | 0.0095 | No |
| 7 | MT1H |  |  | 2140 | 2.422 | 0.0242 | No |
| 8 | PLAG1 |  |  | 2201 | 2.387 | 0.0384 | No |
| 9 | KCNA1 |  |  | 2559 | 2.169 | 0.0285 | No |
| 10 | GNL1 |  |  | 3040 | 1.913 | 0.0074 | No |
| 11 | CYB5B |  |  | 3763 | 1.599 | -0.0345 | No |
| 12 | VEGF |  |  | 3911 | 1.549 | -0.0335 | No |
| 13 | GPX3 |  |  | 4579 | 1.295 | -0.0736 | No |
| 14 | MAP1LC3B |  |  | 4713 | 1.249 | -0.0738 | No |
| 15 | AVPR1A |  |  | 4755 | 1.235 | -0.0672 | No |
| 16 | HTF9C |  |  | 4986 | 1.161 | -0.0755 | No |
| 17 | NEUROD6 |  |  | 5581 | 0.949 | -0.1128 | No |
| 18 | SF3A2 |  |  | 6044 | 0.796 | -0.1414 | No |
| 19 | BIRC4 |  |  | 6051 | 0.794 | -0.1356 | No |
| 20 | EPB41 |  |  | 6428 | 0.672 | -0.1587 | No |
| 21 | C9ORF74 |  |  | 6593 | 0.624 | -0.1662 | No |
| 22 | IL10RA |  |  | 6598 | 0.621 | -0.1616 | No |
| 23 | JARID1C |  |  | 6662 | 0.602 | -0.1616 | No |
| 24 | ROCK1 |  |  | 6704 | 0.585 | -0.1601 | No |
| 25 | ISG15 |  |  | 6857 | 0.533 | -0.1674 | No |
| 26 | HSF1 |  |  | 7062 | 0.463 | -0.1792 | No |
| 27 | TPM3 |  |  | 7148 | 0.436 | -0.1822 | No |
| 28 | INPP5E |  |  | 7393 | 0.347 | -0.1978 | No |
| 29 | ZUBR1 |  |  | 7541 | 0.291 | -0.2066 | No |
| 30 | AQP1 |  |  | 7776 | 0.199 | -0.2227 | No |
| 31 | CREBBP |  |  | 7868 | 0.158 | -0.2283 | No |
| 32 | GLRX |  |  | 8308 | -0.001 | -0.2615 | No |
| 33 | SERPINE1 |  |  | 8947 | -0.283 | -0.3073 | No |
| 34 | ID2 |  |  | 9049 | -0.335 | -0.3123 | No |
| 35 | ZNF76 |  |  | 9135 | -0.376 | -0.3158 | No |
| 36 | ALG12 |  |  | 10349 | -1.060 | -0.3990 | No |
| 37 | PER1 |  |  | 10374 | -1.077 | -0.3923 | No |
| 38 | HSPA1A |  |  | 10471 | -1.135 | -0.3907 | No |
| 39 | C5 |  |  | 10561 | -1.215 | -0.3878 | No |
| 40 | MAP9 |  |  | 10770 | -1.366 | -0.3928 | No |
| 41 | C7 |  |  | 10961 | -1.538 | -0.3951 | No |
| 42 | AHSA2 |  |  | 11114 | -1.678 | -0.3933 | No |
| 43 | GNAI2 |  |  | 11509 | -2.046 | -0.4070 | No |
| 44 | NEU1 |  |  | 11789 | -2.317 | -0.4099 | Yes |
| 45 | GRAMD1C |  |  | 11830 | -2.352 | -0.3944 | Yes |
| 46 | ECHDC2 |  |  | 12007 | -2.566 | -0.3876 | Yes |
| 47 | TSNAX |  |  | 12040 | -2.628 | -0.3693 | Yes |
| 48 | WIPI1 |  |  | 12116 | -2.722 | -0.3536 | Yes |
| 49 | CRYL1 |  |  | 12233 | -2.894 | -0.3397 | Yes |
| 50 | SCYL3 |  |  | 12331 | -2.998 | -0.3235 | Yes |
| 51 | FOSB |  |  | 12385 | -3.084 | -0.3033 | Yes |
| 52 | FOS |  |  | 12389 | -3.085 | -0.2793 | Yes |
| 53 | TRIM52 |  |  | 12737 | -3.709 | -0.2763 | Yes |
| 54 | FLNB |  |  | 12764 | -3.781 | -0.2486 | Yes |
| 55 | CALCOCO1 |  |  | 12786 | -3.834 | -0.2201 | Yes |
| 56 | PIP5K2C |  |  | 12796 | -3.883 | -0.1903 | Yes |
| 57 | BCKDK |  |  | 12859 | -4.025 | -0.1634 | Yes |
| 58 | SPARCL1 |  |  | 12889 | -4.108 | -0.1334 | Yes |
| 59 | RHOB |  |  | 13091 | -4.795 | -0.1109 | Yes |
| 60 | TNCRNA |  |  | 13139 | -4.984 | -0.0753 | Yes |
| 61 | GLI3 |  |  | 13213 | -5.527 | -0.0375 | Yes |
| 62 | RHBDF1 |  |  | 13243 | -5.792 | 0.0058 | Yes |
Table: GSEA details [plain text format]

  

Fig 2: HDACI\_COLON\_TSABUT\_UP: Random ES distribution      
 Gene set null distribution of ES for **HDACI\_COLON\_TSABUT\_UP**
